# Contemporary management of tooth replacement in the traumatized dentition

**DOI:** 10.1111/j.1600-9657.2012.01122.x

**Published:** 2012-06

**Authors:** Aws Alani, Rupert Austin, Serpil Djemal

**Affiliations:** 1 Department of Restorative Dentistry, Newcastle Dental HospitalNewcastle; 2 Department of Prosthodontics, Guy's Hospital, King's College London Dental InstituteLondon; 3 Department of Restorative Dentistry and Traumatology, Kings College HospitalLondon, UK

**Keywords:** diagnosis, permanent tooth, prognosis, tooth injury, treatment

## Abstract

Dental trauma can result in tooth loss despite best efforts at retaining and maintaining compromised teeth (Dent Traumatol, 24, 2008, 379). Upper anterior teeth are more likely to suffer from trauma, and their loss can result in significant aesthetic and functional problems that can be difficult to manage (Endod Dent Traumatol, 9, 1993, 61; Int Dent J 59, 2009, 127). Indeed, teeth of poor prognosis may not only present with compromised structure but trauma may also result in damage to the support tissues. Injury to the periodontium and alveolus can have repercussions on subsequent restorative procedures (Fig. 19). Where teeth are identified as having a hopeless prognosis either soon after the incident or at delayed presentation; planning for eventual tooth loss and replacement can begin at the early stages. With advances in both adhesive and osseointegration technologies, there are now a variety of options for the restoration of edentate spaces subsequent to dental trauma. This review aims to identify key challenges in the provision of tooth replacement in the traumatized dentition and outline contemporary methods in treatment delivery.

## Treatment considerations and the continuum of care

**Fig. 1:**
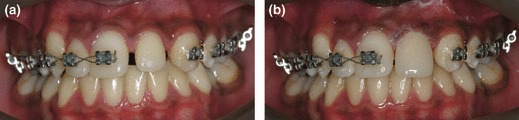
(a) 21-year-old patient who previously lost 21 because of trauma. Orthodontic space optimization was instigated followed by composite augmentation of the 22 to mimic a 21 (b).

**Fig. 2:**
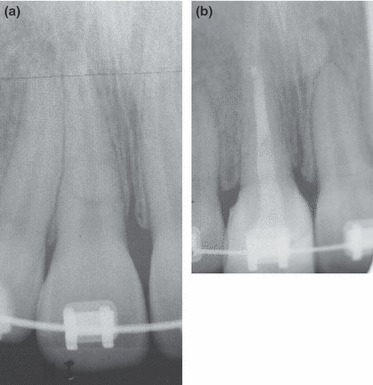
(a) Long cone periapical of 11 following trauma which resulted in mid-root fracture of the 11. (b) The tooth was extracted and an autotransplanted premolar was used to replace. Composite augmentation and root canal treatment was completed on a subsequent visit. Case courtesy of the Department of Paediatric Dentistry, Newcastle Dental Hospital.

Dental trauma can present with severe injuries on multiple teeth that were otherwise unrestored with no history of intervention up to that point. Indeed, when teeth present with multiple concomitant injuries, the relative prognosis of individual units may be difficult to ascertain (4–6). As such, long-term planning may be best carried out after the acute healing phase is complete. In complex cases such as those of polytrauma, multidisciplinary team planning may be sought as treatment options may depend on issues that the primary clinician may not be fully sensitive to. Phased treatment moving from one discipline to another is a regular occurrence where orthodontic treatment is required or where surgical intervention is envisaged (7, 8). These cases may be best planned and reviewed in joint team meetings with agreed strategies for future treatment (9, 10). In the developing dentition, the option of implants may not be available until growth is completed (11). In these cases, an orthodontic opinion may be sought early in the process to assess future development and spacing. Indeed, as alveolar dimensions and gingival maturity are likely to change through adolescence, treatment planning should focus on the definitive option once growth has ceased. This situation requires some foresight as longitudinal planning can optimize outcomes into adulthood (12). The orthodontic-restorative interface may result in treatment options that do not require frank prosthetic tooth replacement. Techniques in orthodontic space closure and restorative augmentation with composite may provide acceptable and serviceable results without the need for tooth tissue removal (Fig. 1) (13). Where tooth positions and stages of root development are favourable autotransplantation of premolar, units have shown excellent long-term results (Fig. 2) (14).

Once post-trauma stabilization has been achieved, an objective assessment of the dentition can commence. Where non-vital teeth have questionable restorability but are amenable to endodontic treatment, the primary aim of preventing the development of an apical lesion and postponing extraction can be considered an astute way of preserving the alveolar form (15, 16). Maintenance of teeth in this way can aide implant provision especially where extraction and immediate placement is envisaged once growth is complete.

Contemporary restoration of the edentate space in the traumatized dentition should ideally be independent of abutment teeth or minimally invasive without compromising mass and quality of tooth structure. Conventional bridgework can result in loss of vitality of abutment teeth, whereas removable partial dentures may result in significant plaque accumulation and also lack social acceptability (17–19). The utilization of minimally invasive techniques may be clinically successful without the need for further intervention.

## Trauma and the resin-retained technique

**Fig. 3:**
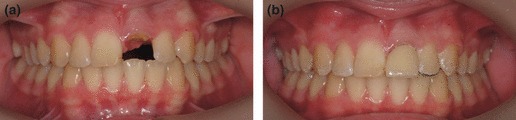
(a) 14-year-old patient presenting with decoronation of the 21 and subluxation of 11. Both teeth were root canal treated and asymptomatic at review. (b) Resin-bonded bridgework cantilevered from the 22 into the 21 space. The guarded prognosis of the 11 precluded it as an abutment. Once growth is completed, the definitive restoration of the 21 area with an implant will be considered.

**Fig. 4:**
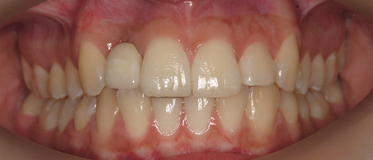
Resin-bonded bridge cantilevered from the 13 into the 12 space. Initial investigations revealed the need for grafting in the 12 site for an implant. The patient was not keen on this option, and a definitive resin bonded bridge was fitted.

The original work by Rochette some 40 years ago on a technique for bonding metal splints to periodontally involved teeth has since undergone significant developments and improvements in addition to new concepts and indications (20). The ability to provide a prosthetic replacement without biologically harmful and irreversible preparations has applications in traumatology especially where immature pulp tissue may be present. This concept is more relevant in the developing dentition where changes in tooth position, alveolar growth and tooth prognosis are still to be realized (21) (Fig. 3). Where teeth have erupted to a level that provides adequate surface area for bonding resin bridges can be delivered. Indeed, their provision can be considered a definitive option where there is inadequate bone volume and quality and where favourable abutment teeth are present (21) (Fig. 4). This option may be more carefully considered by the patient where the need for numerous surgical episodes is required for an osseointegrated restoration, in comparison, the resin-bonded bridge can be delivered sooner with minimal morbidity.

## Preparation or consolidation?

**Fig. 5:**
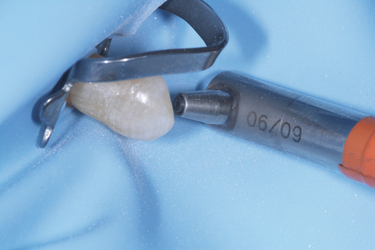
Intraoral sandblasting prior to RBB cementation under rubber dam isolation.

**Fig. 6:**
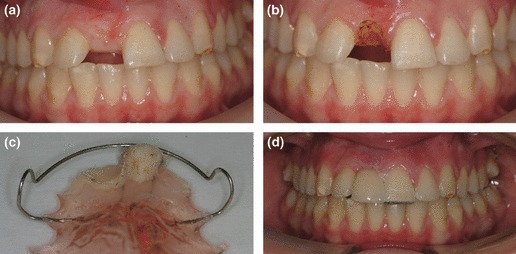
(a) Previous trauma resulted in the loss of the 11. Note the excess tissue in the 11 site making potential pontic dimensions difficult to match with adjacent gingival margins. (b) Electrosurgery to create ideal pontic space for resin-bonded bridgework in addition to maximizing enamel surface area palatally on 21. (c) To maintain the soft tissue dimensions, the retainer was relined and fitted. (d) The definitive resin-bonded bridge cemented from the 21 cantilevered into the 11 space.

The median survival rate for resin bridges has been shown to be just under 10 years with an 87.7% survival rate of bridges and splints at 5 years (22, 23). Features that have been shown to improve survival include surface area covered by the retainer, operator experience and design (24). The extent to which the abutment can be prepared varies (20) and success can be achieved without preparation; although this needs to be balanced against evidence that has suggested improved longevity when retentive features such as rest seats and guide planes are incorporated (24). Newer techniques in optimizing the enamel surface include the use of intra oral sandblasting prior to etching although this has not been evaluated fully (25). This technique may be particularly useful where the tooth surface is extrinsically stained or where residual resin remains from previous bonding attempts. Indeed, the use of bioactive-glass air abrasion that can selectively remove resin as opposed to enamel has also been developed (26) (Fig. 5). Where gingival tissues encroach on the palatal aspect of the potential abutment, this can limit the surface area for bonding and the height of the connector (Fig. 6). These problems can be addressed utilizing electrosurgery to simultaneously augment the prospective pontic site to improve emergence whilst also exposing greater enamel tissue palatally (27). The maintenance of soft tissue postsurgery can be achieved by way of relining a removable prosthesis (Fig. 6c).

**Fig. 7:**
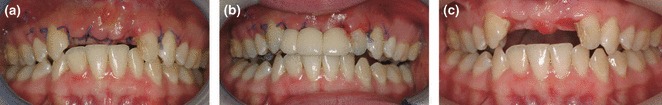
(a) 15-year-old patient who suffered a combination of avulsions, alveolar and root fractures. These were managed surgically and primary closure was achieved. At an early stage, the lack of interocclusal space for subsequent restoration of the edentate space was apparent. (b) To create interocclusal space utilizing the Dahl approach, a resin-bonded bridge was cemented from the 13 to the 22 at an increased occlusal vertical dimension. (c) After 6 months of wear posterior contacts re-established and adequate interocclusal space was created for the placement of implants to definitively restore the space.

Where there is a lack of interocclusal space between potential resin-retained abutments and opposing tooth units, the cementation of restorations at an increased occlusal vertical dimension to create space has been described (28). This technique can prevent the need for tooth preparation and where there is limited prosthetic envelope for future restorations, space can be created for definitive planning (Fig. 7). Axial tooth movements vary between individuals; younger patients have a greater scope for a combination of intrusion and eruption, whereas in older patients, the movements are predominantly intrusion (29).

## New modalities

**Fig. 8:**
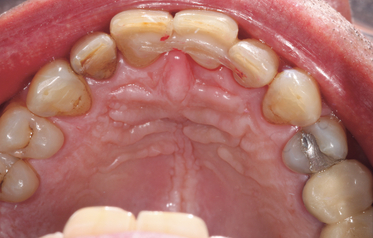
Immediate resin-bonded bridgework for 21 space utilizing carbon fibre material.

Technological developments in adhesive technology have resulted in the ability to utilize the crowns of avulsed teeth as pontics in an immediate manner (Fig. 8) (30). Further to this, the development of fibre-reinforced resin-bonded bridges has presented clinicians with greater choice when considering minimally invasive options for tooth replacement (31). These materials have been described as resin-based restorations containing fibres aimed at enhancing their physical properties (32). As the framework is tooth coloured, aesthetic problems relating to show through of abutments can be minimized (32). There is potential for development of the bond of the cement lute to the retainer wing being stronger than that to metal because of greater linearity between the materials. The dentist has the choice of fabricating the prosthesis directly (if for example an avulsed tooth is available to be modified) or indirectly (Fig. 8). One consideration is the need for greater occlusal clearance required for the retainers, and cantilever designs may not be achievable because of the lack of rigidity.

**Fig. 9:**
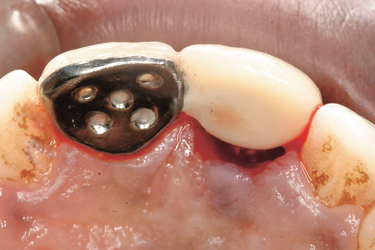
Rochette style bridge utilized in a multiphase treatment plan. This patient was fitted with the resin-bonded bridge immediately after extraction. The bridge was modified to accommodate a healing abutment and recemented after implant placement.

A practical advantage of resin-bonded bridges is their retrievability, especially where a multiphase treatment plan is envisaged (Fig. 9). Initial cementation post-trauma may provide an interim measure until growth is completed or the dentition fully stabilized. Resin-bonded bridges lend themselves to this ethos as removal is relatively straightforward by ‘tapping off’ the bridge when needed. The use of glass ionomer cement as opposed to resin composite has also been recommended where removal is envisaged at a later date.

## Utilization in multi-phased treatment

Banerji and colleagues examined the use of RBBs as an interim restoration during implant treatment (33). The study examined two phases in the use of the Rochette bridge, the first after immediate cementation following extraction and the second at the time of implant surgery and recementation. In the first phase, 16% of bridges required recementation in comparison with 27.5% and in the second phase, 7.2% of bridges required recementation in both phases (33). Interestingly, there was marginal difference in probability of survival between the two phases over 200 days with phase one being 80% and phase two 78%. This study provides some scope for RBB use in the longitudinal treatment of the traumatized dentition (33).

## Limitations of the technique

Resin-retained bridges have disadvantages that may result in the consideration of alternative options. The reported survival rates may be less than alternative, albeit more invasive options, such as conventional bridges or implants (23). Indeed, the degree of patient satisfaction with resin-bonded bridges appears to be high and does not seem to be influenced by the occurrence of failure or the possibility of recementation (34). Provision of RBBs retained by thin anterior teeth may result in metal show through which may not be acceptable to some patients. This can be minimized by use of an opaque cement (20). Try-in of the restoration is difficult due to absence of retention without cementation. This can be remedied using calcium hydroxide-based lining materials to temporarily fixate the bridge *in situ* to assess aesthetics (35). Occlusal control may be difficult to achieve because of the encroachment on previous anterior guidance pathways, in cases of poly-trauma, potential abutment teeth may be compromised precluding them as suitable abutments. In cases where multiple adjacent teeth are lost, the scope for the provision of resin-retained bridgework is limited. Indeed, if occlusal factors and abutment teeth are unfavourable, the clinician must consider dental implants as an alternative fixed treatment option.

## Osseointegration in the traumatized dentition

### Managing the aftermath of trauma

**Fig. 10:**
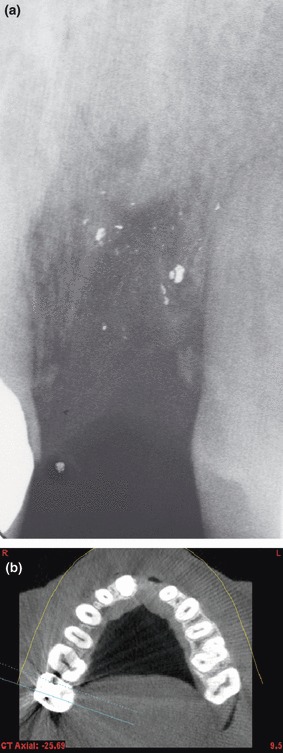
(a) This patient suffered trauma to the 21 which was subsequently treated with orthograde endodontics followed by apical surgery. Because of persistent infection, the tooth was extracted. (b) CBCT examination revealed an obvious bony defect which was not amenable to implant placement. The feasibility of bone graft placement was also difficult to predict because of the lack of bone present to receive donor tissue.

**Fig. 11:**
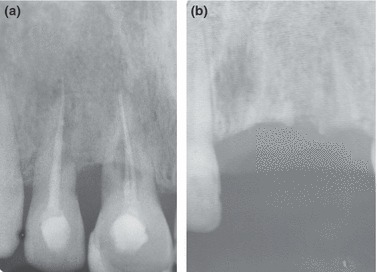
(a) Radiographic examination of the 11 and 12 showed external and internal inflammatory resorption and external replacement resorption. (b) The 11 and 12 were accessed and gutta percha removed and were subsequently decoronated with surgical closure.

**Fig. 12:**
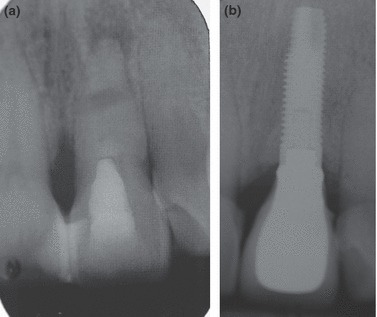
(a) Delayed presentation of a mid-root fracture and a periapical lesion. (b) The 21 was replaced with an implant.

**Fig. 13:**
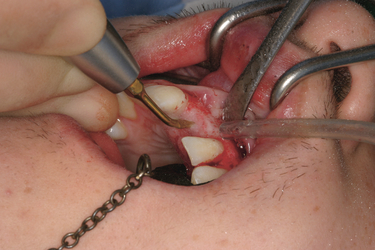
Piezo powered bone and periodontal ligament dissection.

Planning for implants in the trauma patient can be challenging as well as clinically difficult particularly when the extent of trauma is directly related to the feasibility of treatment (Fig. 10). Postextraction changes can result in buccal bone loss making implant provision more difficult (36). Attempts at maintaining hard and soft tissue topography have included atraumatic extraction techniques in combination with adjunctive tissue regeneration (37). In comparison, the pathophysiology of dental trauma with the possibility of a superimposed endodontic infection fuelling the resorptive process results in a more aggressive and rapid loss of bone and soft tissue contour. Ankylosis and progressive bone or root resorption can further complicate treatment. As a result, techniques of elective decoronation have been described with the aim of preventing infraocclusion and allowing alveolar development to continue (38–40) (Fig. 11). Where horizontal fractures present, the extraction of both fragments or simply the most coronal portion needs to be considered (Fig. 12). Attempts at retrieval of an apical fragment may result in the loss of further bone because of restricted access. In contrast, retention of the fragment and its removal at the implant placement stage although desirable may be difficult to achieve. This remains an area where the clinician’s best judgment in retaining as much bone as possible for implant placement needs to be balanced against the need for fragment removal. Recent innovations in atraumatic exodontia have included the use of implant drills to thin root walls prior to implant placement and the use of special elevators and modified piezo tips for dissecting the periodontal ligament (41–43) (Fig. 13). The difficulty in dealing with such cases may require input from surgical colleagues in the execution of adjunctive procedures for subsequent implant placement.

### Optimization of the edentate site

**Fig. 14:**
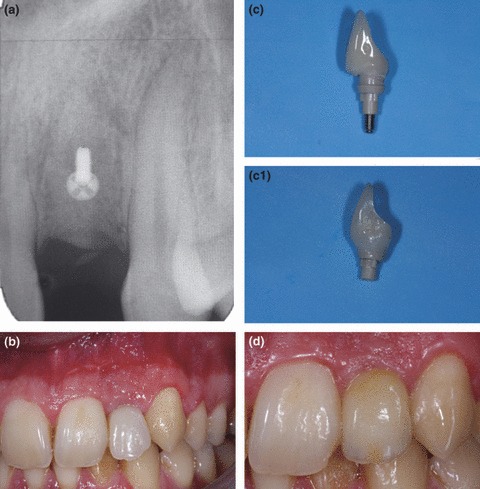
(a) Onlay graft in the 22 site where previous avulsion had resulted in deficient alveolar profile for implant placement. (b) Interim implant restoration 22. Note the lack of emergence. (c) Flowable composite modification of the interim restoration to create gradual emergence and form. (d) The definitive restoration with developed emergence form.

Where multiple teeth are lost or alveolar bone has resorbed following trauma, the need for adjunctive bone grafting prior to provision of implants may need to be considered. Indeed, the clinical appearance may not reveal the true extent of the bone deficit because of hypertrophy of the mucosa (Fig. 10). The assessment of the edentate site can be evaluated using a variety of techniques such as clinical ridge mapping or cone beam–computerized tomography (44). Where there is a lack of bone width in the presence of adequate vertical height, onlay grafting can be utilized (Fig. 14). This can establish bucco-palatal bone width but also improves the scope to place the implant in the appropriate axial and mesio-distal position. The options for donor sites include the ascending ramus, the anterior mandible or an extra-oral site. Adjunctive procedures are not without complications; partial or total block graft failure has been documented at 7% and 8%, respectively (45). In contrast, soft tissue complications were more common ranging from membrane exposure (30%), incision line opening (30%), perforation of the mucosa over the graft site (14%) and infection of the graft site (13%) (45).

**Fig. 15:**
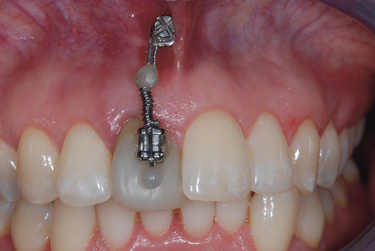
Patient undergoing orthodontic extrusion for the purpose of implant site development in the 11 site. The 11 has been extruded utilizing a mini implant placed apically. Case courtesy of Bill Ip, Newcastle Dental Hospital.

Forcible orthodontic eruption of an otherwise unrestorable lateral incisor for the purposes of alveolar bone development has been described (46) (Fig. 15). In cases where vertical augmentation is required, distraction osteogenesis can be considered either for a single or multiple unit sites. The movement of arch segments by way of distraction osteogenesis has been described for implant site optimization (47). Distraction devices placed after osteotomy preparation can be transalveolar or extra-alveolar and can require surgical fixation. A latency period of 1 week prior to commencing distraction at the rate of 0.5–1 mm a day has been recommended although this will depend on the individual case and the magnitude of distraction required (48).

### The importance of support tissue profile

**Fig. 16:**
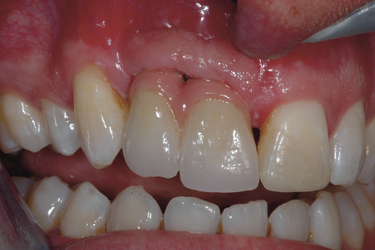
Traumatic loss of 11 and 12 resulted in a marked vertical and horizontal defect. The patient preferred the use of gingivally toned ceramic as opposed to bone and soft tissue grafting prior to implant placement. Case courtesy of Amre Maglad, Newcastle Dental Hospital.

Where adequate alveolar mass and gingival biotype is present, the provision of implant restorations anteriorly can be predictable providing adequate aesthetics and function. Such provision may be more complicated when the loss of two adjacent teeth is present. Where bone volume is adequate but soft tissues are deficient, the clinician may consider the use of graft procedures to optimize soft tissue coverage. Alternatively, the patient may be keen on an implant-based restoration without the need for adjunctive grafting. The option of gingivally toned ceramic as an alternative to vertical augmentation and soft tissue grafting has been recently highlighted (49) (Fig. 16).

The gingival component of a restoration is important especially where osseointegration is planned. The aesthetics of the gingiva associated with a restoration has been shown to be a factor in the success of the restoration (50). Patients with a thicker biotype have shown more favourable long-term results than those with thinner soft tissues (51). In a recent study of 513 patients presenting with orofacial trauma, 29.2% had signs of either gingival or oral mucosa injuries (52). Both biotype and previous trauma injuries may affect the degree to which these tissues can be manipulated. Techniques in the creation of a gradual natural emergence profile of implant restorations have been described (53, 54) (Fig. 14). These techniques aim to manipulate the peri-implant tissues to create a more natural emergence. Foresight in the management of soft tissues in acute trauma by optimizing healing may improve the scope for tissue manipulation for future restorations.

### Timing of implant placement

**Fig. 17:**
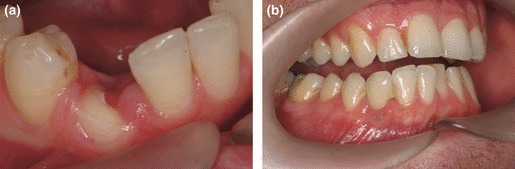
(a) Decoronation of 43 as a result of trauma. Because of the limited supragingival tissue available for restoration, the tooth was deemed as having a poor prognosis. (b) Definitive restoration after extraction and immediate implant placement.

The various timings of implant placement postextraction have recently been investigated (55). A systematic review comparing the outcomes of immediate, immediate delayed and or delayed implants suggested that immediate and immediate delayed placement came with a higher chance of postoperative complications although immediate placement may present with better aesthetic outcomes (55) (Fig. 17). Despite these conclusions, the authors felt there is a need for better quality of evidence as the current literature is sparse, underpowered and carried a high risk of bias (55). More specific studies have examined the outcomes of implants placed in extraction sites of teeth with periapical lesions (56–58). One systematic review concluded that the immediate approach may require thorough debridement of the extraction socket, prophylactic antibiotics, tissue regeneration and in some cases result in impaired bone to implant contact (58). Other controlled studies have shown favourable results with limited complications and survival up to 3 years (56, 57). As long-term outcome studies are lacking, the predictability and longevity of placing implants immediately into extracted sites is still to be realized. Where teeth are avulsed in an acute trauma situation, the clinician may be faced with the option of placing an implant into a recently traumatized site. This decision can be difficult as the uncertainties of prognosis of adjacent teeth and the status of bone in the avulsion site itself can be hard to judge. Immediate implant placement reduces the number of surgical episodes and treatment time. The procedure may be technique sensitive, there may be a lack of keratinized tissue available for flap adaptation, and the site morphology may complicate optimal placement. In contrast, the delayed immediate approach at 4–8 weeks which will have increased keratinized soft tissue available in addition to assessment of any developing pathologies. Unfortunately, the healing site may have already undergone significant resorption by the time the implant is placed.

The implant provision pathway in trauma patients has been examined in a retrospective study (59) where implants were placed in 42 sites between 6 months to 11 years post-trauma. In 17% of cases, there was a deficiency of bone that required adjunctive procedures. Four of the 42 implants exhibited postsurgical complications, whereas five exhibited complications after cementation of the crown. One significant finding was that patients who had long-standing tooth loss required grafting. In addition to this, all patients who had lost two or more adjacent teeth also required grafting. One may speculate as to whether immediate implantation following tooth loss may have prevented the need for grafting.

This view may be strengthened by a more recent retrospective study of 53 trauma patients provided with implants. Eighty-one per cent required bone augmentation that included onlay grafts or guided bone regeneration and 47% underwent immediate placement and in some cases with immediate loading (60). The authors found a 45% complication rate, although complications were significantly less in those cases with no previous history of periapical pathology. The results of this study may strengthen the case for remedial root canal treatment to decrease the presence of inflammation and potentially increase bone for apical engagement of the implant.

### Long-term maintenance of implants in the trauma patient

**Fig. 18:**
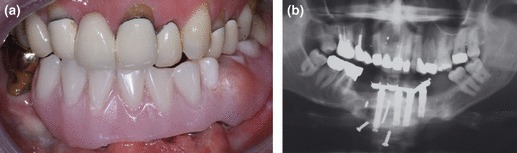
(a) This patient suffered numerous injuries subsequent to a road traffic accident approximately 25 years ago. She lost the majority of her mandibular teeth in addition to the need for grafting and fixation. Once stabilized, she was provided with an implant retained prosthesis. (b) Radiograph 20 years after initial provision. During this period, the patient presented with a variety of maintenance requirements that included clip fractures, abutment screw loosening, overdenture replacement and peri-implant mucositis.

**Fig. 19:**
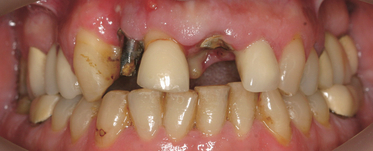
Poly-trauma presenting with decoronation of the 21, luxation of the 11, fracture of the implant crown 12, gingival lacerations and a sinus associated with the 11.

As the majority of traumatic injuries occur in the young or adolescent patient, the restorative dentist needs to consider the longevity and serviceability of the restoration (Figs 18 and 19). Indeed, if definitive tooth replacement therapy is provided soon after growth is completed, the requirement for future maintenance and restoration replacement needs to be considered. Furthermore, ongoing care is likely to be of great significance in future as our patients are living for longer (61).

Biological complications such as peri-implantitis have been reported to be as high as 56% (62). Mechanical complications such as abutment screw fracture or loosening also need consideration. Mechanical complications may be more likely in a patient who is susceptible to trauma. Stuebinger and colleagues reported the bending of abutment screws in a patient who sustained trauma to his implant restorations (63). Allen and Allen (64) reported the fracturing of inter-implant bone subsequent to a blow to the face which also resulted in abutment screw bending. Flanagan (65) reported the fracturing of implant crowns when soft tissue injuries were sustained. It would seem sensible to consider the likelihood of repeat trauma and its repercussions on tooth replacement and subsequent maintenance when managing this group of patients.

## Conclusion

The provision of tooth replacement in the traumatized dentition has specific challenges that may not be present in patients who have suffered plaque-related tooth loss. This can make the treatment planning process more difficult requiring adjunctive procedures to aid the definitive result.
